# Tumor-associated macrophages (TAMs): Constructing an immunosuppressive microenvironment bridge for pancreatic ductal adenocarcinoma (PDAC)^☆^

**DOI:** 10.1016/j.cpt.2024.07.004

**Published:** 2024-07-23

**Authors:** Runjie Liu, Jianang Li, Liang Liu, Wenquan Wang, Jinbin Jia

**Affiliations:** a Department of Pancreatic Surgery, Zhongshan Hospital, Fudan University, Shanghai 200032, China; b Department of Basic Medicine, Shanxi Medical University, Taiyuan, Shanxi 030000, China; c Department of Basic Medicine and Institute of Liver Diseases, Shanxi Medical University, Taiyuan, Shanxi 030000, China; d Cancer Center, Zhongshan Hospital, Fudan University, Shanghai 200032, China; e Department of General Surgery, Zhongshan Hospital, Fudan University, Shanghai 200032, China

**Keywords:** Pancreatic cancer, Macrophages, Tumor microenvironment, Immunosuppression

## Abstract

Pancreatic ductal adenocarcinoma (PDAC) is a fatal disease with increasing incidences worldwide. The overall 5-year survival rate remains low, underscoring the urgent need for effective therapies. Despite the promising efficacy of immunotherapy for various solid tumors, its benefits for pancreatic cancer have been disappointing. This is largely because of the complex and unique mechanisms of immune evasion inherent in PDAC. Emerging evidence has highlighted the pivotal role of tumor-associated macrophages (TAMs) in facilitating the immune escape of PDAC. TAMs significantly contribute to forming an immunosuppressive microenvironment, which hinders the effectiveness of immunotherapeutic approaches. They achieve this through multiple pathways, including the secretion of cytokines and the promotion or inhibition of multiple immune cells. In this review, we summarized the main pathways through which TAMs form an immunosuppressive microenvironment in PDAC. We also examined the current status and recent progress of immunotherapy strategies that specifically target macrophages. By understanding these mechanisms and exploring targeted therapies, we aimed to shed light on potential avenues for improving the treatment outcomes of this devastating disease.

## Highlights

• Tumor-associated macrophages (TAMs) have the ability to promote and inhibit pancreatic ductal adenocarcinoma (PDAC).
• Crosstalk between TAMs and immune cells is the key to an immunosuppressive microenvironment.
• Targeting TAMs for reverse immunosuppression is a future direction of PDAC treatment.

## Graphical abstract

**Figure:**
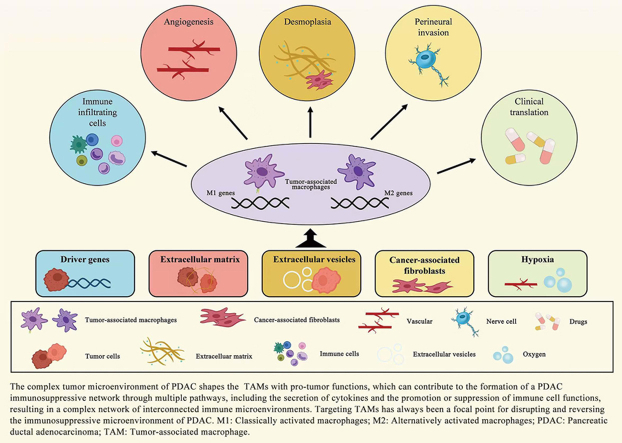

## Introduction

Pancreatic ductal adenocarcinoma (PDAC) is a highly lethal disease that is expected to become the second leading cause of cancer-related deaths by 2030.^1^ The 5-year survival rate of PDAC is only 13%,^2^ and about 80–85% of the patients have unresectable or metastatic disease.^3^ Combined chemotherapy with FOLFIRINOX and gemcitabine/nab-paclitaxel can improve the survival rate; however, this is still far from satisfactory.^4^^,^^5^ Therefore, a more systematic systemic treatment is required to alleviate the current situation.

**Figure 1:**
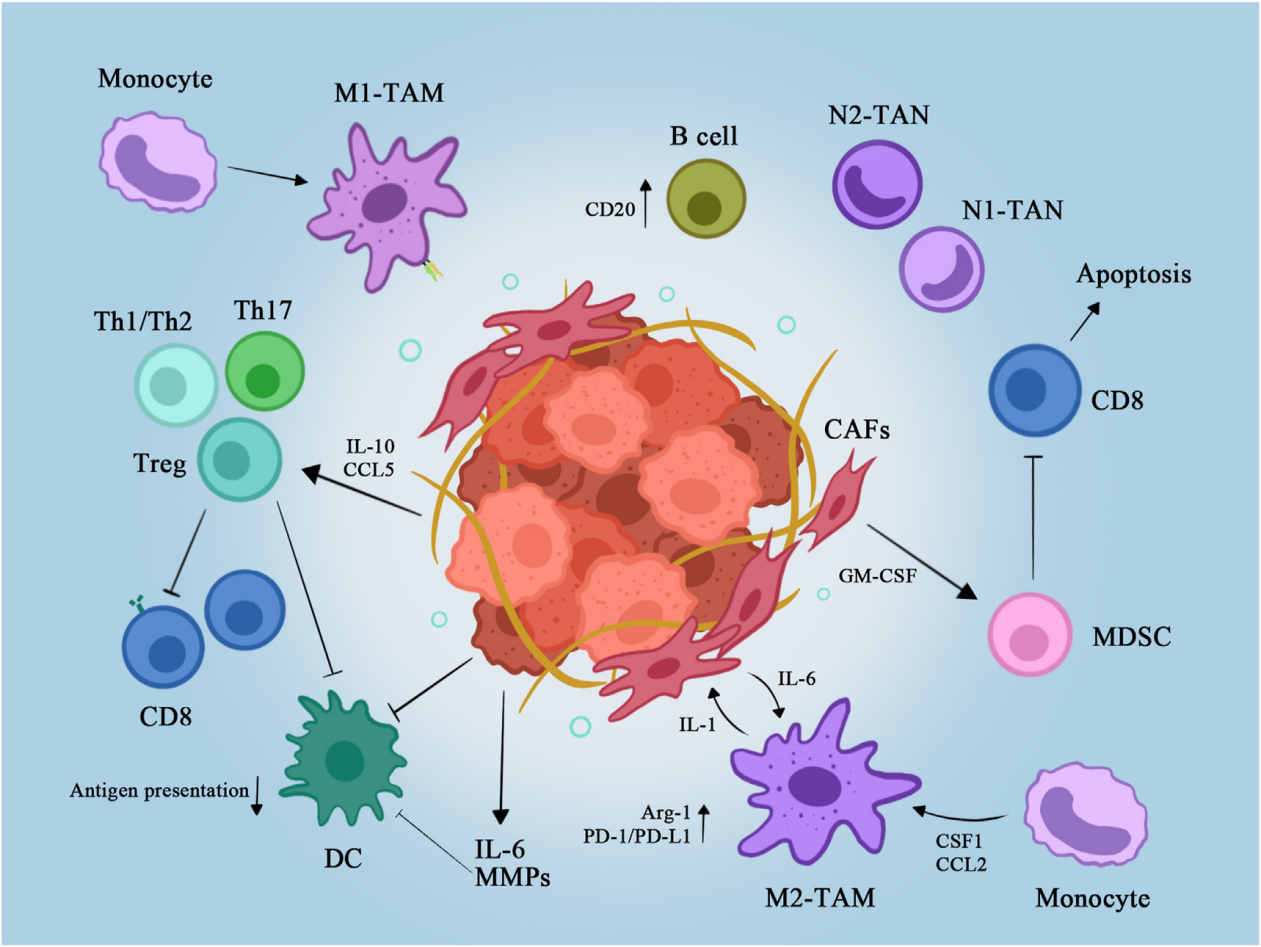
Immunosuppressive properties of PDAC. Cancer is caused by tumor-related genetic abnormalities, with consequent cascading changes in the immune microenvironment. Arg-1: Arginase-1; CAF: Cancer-associated fibroblast; CCL2: CC chemokine ligand 2; CD: Cluster of differentiation; CSF1: Colony-stimulating factor 1; DC: Dendritic cell; GM-CSF: Granulocyte-macrophage colony-stimulating factor; IL: Interleukin; M1: Classically activated macrophages; M2: Alternatively activated macrophages; MDSC: Myeloid-derived suppressor cell; MMP: Matrix metalloproteinase; PD-1: Programmed death 1; PDAC: Pancreatic ductal adenocarcinoma; PD-L1: Programmed death ligand 1; TAM: Tumor-associated macrophage; TAN: Tumor-associated neutrophil; Th: T helper; Treg: Regulatory T cell.

The development of immunotherapy offers a glimmer of hope for a variety of refractory malignancies, showing efficacy against metastatic melanoma, renal cell carcinoma, and non-small cell lung cancer.^6^^,^^7^ However, the results were mostly disappointing following the application in PDAC.^8^ PDAC is a low-immunogenic tumor in which it is difficult to produce and release new antigens, resulting in low levels of tumor-infiltrating lymphocytes (TILs).^9^ Immunosuppressive cells in the PDAC microenvironment, such as tumor-associated macrophages (TAMs), tumor-associated neutrophils (TANs), cancer-associated fibroblasts (CAFs), myeloid-derived suppressor cells (MDSCs), and regulatory T cells (Tregs), together form a unique immunosuppressive microenvironment that constitutes a barrier to immunotherapy^10^ [Figure 1].

Macrophages are key components of the innate immune system that express cytotoxic T-lymphocyte-associated protein 4 (CTLA4), programmed cell death protein 1 (PD-1), programmed cell death ligand 1 (PD-L1), and other adaptive immune checkpoint molecules. The complex immune functions of macrophages indicate that they play a pivotal role in PDAC immunity. TAMs are one of the main infiltrating immune cells that act as bridges for constructing the immunosuppressive microenvironment of PDAC; regulating TAMs may restore the anti-tumor immunity of PDAC. This paper reviews the roles and underlying mechanisms of TAMs in PDAC immunity and briefly reviews the therapeutic strategies for targeting macrophages.

## The recruitment and polarization of tumor-associated macrophages

PDAC is characterized by a low tumor cell content, abundant stroma, and a lack of vasculature. Prominent infiltration of immunosuppressive cells into the tumor microenvironment (TME) has been observed during the development of PDAC.^11^ Macrophages are responsible for constructing an immunosuppressive TME in PDAC.^12^ TAMs in PDAC have heterogeneous origins, with embryo-derived macrophages comprising the majority, followed by monocytes. Macrophages derived from embryo progenitor cells proliferate in PDAC tissues and show a profibrotic phenotype, whereas monocyte-derived macrophages may play more potent roles in regulating adaptive immunity because of the higher levels of messenger ribonucleic acid (mRNA) involved in class I and class II antigen presentation.^13^ TAMs are considered heterogeneous populations in a state of constant transition between the two forms of M1 and M2 types.^14^ Here, M2-type macrophages are used to summarize the classes of macrophages with tumorigenic effects.

### Heterogeneity and polarization of tumor-associated macrophages

Although there is currently a consensus that TAMs can be divided into two subtypes according to their surface molecules, M1-like and M2-like TAMs, the complexity of macrophage polarization goes far beyond this binary classification. Pro-inflammatory macrophages (M1-like) are considered to have anti-tumor effects in tumors, and anti-inflammatory macrophages (M2-like) generally show pro-tumor effects. Under the control of different driver genes, a large and complicated microenvironmental network consists of an acellular extracellular matrix (ECM), multiple cell-released growth factors, chemokines, and cytokines that synergistically control the functional transformation of macrophages.

#### M1-subtype

The interaction of the interferon-gamma receptor (IFN-γR), granulocyte-macrophage colony-stimulating factor receptor (GM-CSFR), and interleukin-12 receptor (IL-12R) on macrophages with their ligands leads to M1-like polarization through the Janus kinase (JAK)/signal transducer and activator of transcription (STAT) pathway.^15^^,^^16^ IFN-γR mediates macrophage function mainly through the JAK1/STAT1 pathway, while IL-12R commonly goes to the JAK2/STAT4 pathway. GM-CSFR triggers not only the JAK2/STAT5 pathway but also the nuclear factor kappa B (NF-κB) and phosphatidylinositol 3-kinase (PI3K)/protein kinase B (AKT) signaling pathways that favor pro-inflammatory polarization of macrophages.^15^^,^^17^ As one of the pattern recognition receptors (PRRs), Toll-like receptors (TLRs) are deregulated in several cancers.^18^, ^19^, ^20^ TLRs are involved in myeloid differentiation primary-response protein 88 (MyD88)-dependent and TIR-domain-containing adapter-inducing interferon-β (TRIF)-dependent signaling pathways that promote polarization to M1 macrophages.^15^

The tumor necrosis factor (TNF) receptor family plays a role in the release of pro-inflammatory factors by activating classical NF-κB signaling in macrophages.^21^^,^^22^ The popular receptor from the TNF receptor (TNFR) family, co-stimulatory receptor CD40, has attracted much attention for the treatment of PDAC.^23^, ^24^, ^25^, ^26^ CD40, expressed on macrophages, interacts with ligands on activated T or B cells, leading to macrophage activation and the production of nitric oxide (NO).^27^ CD40 activation can trigger fatty acid oxidation and glutamine metabolism to promote adenosine triphosphate (ATP) citrate lyase-dependent epigenetic reprogramming of pro-inflammatory genes and antitumorigenic phenotypes in macrophages.^28^ The CD40 activated-TAMs usually up-regulate major histocompatibility class II (MHC-II), human leukocyte antigen-DR (HLA-DR), the co-stimulatory molecule CD86, and down-regulate CD163 which coincide with elevated serum levels of IL-12, TNF-α, and IFN-γ.^29^^,^^30^

#### M2-subtype

CSF-1 activates the PI3K/Akt and mitogen-activated protein kinase (MAPK) signaling pathways in macrophages^31^ to promote M2-like differentiation, while IL-6,^32^ heat shock protein 90 alpha (HSP90α), etc. promote M2 differentiation via the NF-κB and JAK/STAT3 pathways.^33^ M2-like TAMs are capable of secreting transforming growth factor β (TGF-β), which in turn also promotes the M2-like phenotype via Snail signal transduction.^34^ The TGF-β receptor on the macrophages binds to TGF-β and then phosphorylates, activating Smad2/3 that attaches to Smad4 and resulting in the expression of M2 marker arginase-1 (Arg-1).^35^ However, the regulatory function of TGF-β on macrophages in PDAC depends not just on the Smad pathway, but also on the PI3K or MAPK signaling pathway.^36^^,^^37^ Recent studies have shown that the signal-regulatory protein α (SIRPα) signaling pathway can inhibit the M1-like polarization pathway in macrophages.^38^ Notch signals can also partially change the polarization of macrophages by controlling the expression of SIRPα.^39^

#### Limitations of M1 and M2 tumor-associated macrophages

Traditional M1 and M2 dichotomies do not completely summarize the tissue specificity and stress response of TAMs. In PDAC, some TAMs highly express the M1-associated marker HLA-DR; however, approximately 23.3% of TAMs concomitantly express both the HLA-DR and M2-associated marker CD163.^40^ Collagen-internalized macrophages in PDAC express Arg-1 and inducible nitric oxide synthase (iNOS), a phenotype that simultaneously acquires M1- and M2-related markers.^41^ Immunodynamic analysis in the mouse PDAC model showed that the infiltration of immunosuppressive TAMs is significantly different in pancreatic intraepithelial neoplasia (PanIN) and early tumor stages, with increased initial infiltration appearing to be phenotypic in favor of the tumor suppression function. In the process of pancreatic tissue developing towards the early tumor, the infiltration of macrophages continues to occur^12^ and its function gradually shifts with mutation accumulation and changes in the microenvironment to form dynamic changes.

With the advent of new technologies, such as single-cell transcriptomic sequencing, researchers have discovered that TAMs can be divided into subgroups with distinct characteristics and phenotypes instead of binary differentiation. Using single-cell analysis, Chen et al^42^ identified two additional macrophage subgroups marked by CD169 and T cell receptor (TCR) based on the original M1 and M2 classifications. TCR^+^ macrophages are more abundant in patients with advanced PDAC. Werba et al^43^ identified two distinct macrophage subpopulations marked by secreted phosphoprotein 1 (SPP1), complement component 1, Q subcomponent, and C chain (C1QC). While C1QC^+^ TAMs exhibited a higher M1 characteristic expression compared to SPP1^+^ TAMs, both the SPP1^+^ and C1QC^+^ subsets demonstrated strong M2 characteristic expression. Consequently, these subpopulations cannot be clearly distinguished using the traditional M1/M2 classification system. Dong et al^44^ identified a novel immunological resident tissue macrophage population specific to the paratumor tissue in pancreatic cancer. This macrophage subgroup is characterized by reduced levels of *GLUL* and *SQSTM1* relatively scarce in tumor samples, and exhibits a positive correlation with CD8^+^ T cells. Additionally, several genes that promote tumor progression and metastasis were absent in this subgroup. Consequently, such subgroup may act as a positive regulator of immune responses. Caronni et al^45^ identified a macrophage subpopulation characterized by IL-1β expression, induced through the local synergistic interaction between prostaglandin E2 (PGE2) and TNF. These macrophages are linked to inflammatory reprogramming and acquisition of pathogenic properties, resulting in persistent transcriptional changes that drive disease progression and poor patient outcomes. In contrast, a specific subtype of lipid-associated macrophages (LAMs) plays a crucial role in the liver metastasis of PDAC. These macrophages not only express the traditional macrophage marker CD68 but also show an elevated expression of lipid metabolism genes.^46^ In conclusion, the classical M1/M2 classification system is no longer adequate for characterizing macrophages within the pancreatic cancer TME. However, no precise scientific categorization exists for defining macrophages with varying characteristics. This underscores the complexity of the pancreatic cancer immune microenvironment and highlights the challenges in developing specific targeted therapies.

### Driver genes in pancreatic cancer cells influence tumor-associated macrophages' polarization

Oncogenic pathways in tumor cells can modulate the systemic immune landscape,^47^ and *vice versa*; cytokines from the immunosuppressive microenvironment drive metabolic reprogramming in cancer cells, leading to tumor progression.^48^

#### KRAS

*KRAS* mutations are important contributing factors to the immunosuppressive microenvironment of PDAC. More than 90% of PDAC harbor *KRAS* mutations, with >80% of single-point mutations in the G12 codon; most substitutions occur in *G12D* (∼36%), *G12V* (∼35%), and *G12R* (∼13%).^49^ Co-culturing with *KRAS*^*G12D*^ mutant pancreatic epithelium cells shifts the macrophages toward M2, with an increase in the M2/M1 ratio.^50^ KRAS^G12D^ enhanced the expression of GM-colony-stimulating factor (GM-CSF) in a mouse model of PDAC, resulting in Gr1^+^CD11b^+^ myeloid cell expansion. GM-CSF polarizes TAMs into the M2-type through the PI3K-AKT signaling pathway.^17^^,^^51^ KRAS^G12D^ released during oxidative stress-induced autophagy-dependent cell death in PDAC is absorbed by TAMs, which are then converted into M2-like TAMs via STAT3-dependent fatty acid oxidation.^52^ In contrast, altered metabolic pathways in *KRAS* mutant macrophages lead to the upregulation of metabolic enzymes and expression of M2-like metabolic markers such as Arg-1.^17^

#### TP53

*TP53* inactivation is a relatively late event in PDAC.^53^ In a *KRAS*^*G12D*^-driven PDAC mouse model, loss of *TP53* enhanced the recruitment of immunosuppressive TAMs.^54^ Datta et al^55^ found that compared to KRAS-altered/TP53 WT tumors, KRAS-TP53 co-altered samples exhibited significant increases in TANs and TAMs. There was a concomitant reduction in both global and cytotoxic CD8^+^ T cell populations, specifically with an increase in F4/80^+^CD206^+^ M2-like macrophages but not in F4/80^+^CD86^+^ M1-like macrophages, in KRAS-TP53 co-altered tumors. P53 (encoded by *TP53*) participates in immune checkpoint regulation and cytokine production.^56^ The expression of p53-induced death domain1α (DD1α) on macrophages is required to engulf apoptotic cells.^57^ When p53 was inactivated, Zheng et al^58^ showed that the production of the inflammatory cytokines IL-1, IL-6, and IL-12 by macrophages was enhanced. p53 loss also increases the expression of myeloid attractant CC chemokine ligand 2 (CCL2) and promotes the recruitment of CCR2^+^ macrophages.^59^, ^60^, ^61^ Therefore, such chemokine changes cause an association between higher overall macrophage density and *TP53* alterations.^62^

#### Smad4

*Smad4* inactivation, like *TP53*, occurs rather late in PDAC formation. The SMAD4 protein is part of the TGF-β pathway which is considered a tumor suppressor. Smad4 loss results in a reduction in lymphocyte infiltration, lower T cell marker expression, and decreased T cell-mediated cytotoxicity.^63^ In contrast, Xiong et al^64^ observed a substantial infiltration of CD3^+^, CD4^+^, and CD8^+^ cells into Smad4 KO tumor tissues. *Smad4* deficiency enhances intratumoral dendritic cell (DC) activation by promoting spontaneous DNA damage and stimulating the stimulator of interferon genes (STING)-mediated type I IFN signaling, which helps control tumors. There are several possible reasons for this discrepancy. *Smad4* may play various roles in tumor development at different stages and within different mutational contexts, resulting in significant individual variations. In contrast, Smad4 is transmitted between cells via exosomes. Such Smad4-containing PDAC exosomes construct an immunosuppressive TME by increasing calcium flux and glycolytic activity pathways to recruit MDSCs.^65^ In prostate cancer, low *Smad4* expression is associated with a significantly increased infiltration of memory B cells, CD8^+^ T cells, Tregs, and M2-type macrophages^66^; while in PDAC, the direct effect of *Smad4* mutations on macrophages has not been clearly studied, although Smad4 plays a key role in the recruitment of immuno-infiltrating cells.^67^

### Components of the tumor microenvironment influence tumor-associated macrophages’ polarization and functions

#### Extracellular matrix

PDAC is characterized by heavy ECM deposition, with the main structural proteins being type I, III, and IV collagens.^68^ Both the primary and metastatic sites of human PDAC show marked bridging membrane hyperplasia and increased expression of ECM components such as hyaluronic acid (HA) and collagen,^69^ whereas the tumor-associated ECM plays a fundamental role in determining the phenotype and function of tumors and stromal cells.^70^ A key step in ECM remodeling is collagen degradation; M2-like macrophages can degrade collagen through the mannose receptor (mannose receptor C-type 1 [MRC1]) pathway,^71^ leading to the accumulation of intracellular arginine and a unique Arg1^+^iNOS^+^ phenotype.^41^ The collagen thickness in the ECM can also affect macrophages. The combination of TGF-β-induced protein (βig-h3) and type I collagen forms thick fibers, leading to the high expression of CD61 and activation of the focal adhesion kinase (FAK) signaling pathway in macrophages, which polarizes the macrophages toward the M2 type.^72^ Galectin 4 in the ECM is produced by PDAC cells, and its decrease results in a higher proportion of M1 macrophages, myofibroblast CAFs, T cells, and DCs.^73^ In addition, PDAC matrix metalloproteinase (MMP)-cleaved Col I (cCol I) and intact Col I (iCol I) have opposing effects on macrophage phagocytosis, tumor growth, and metastasis. cCol I activates the discoid DDR1-NF-κB-P62-NRF2 signaling pathway to promote growth, while iCol I inhibits it.^74^ Taken together, the high density and integrity of the stroma affect macrophage polarization and function, suggesting that stromal depletion modulates TAMs to reverse the immunosuppressive microenvironment, but there is an increased risk of tumor metastasis from stromal depletion therapy.^75^ Proteoglycans and HA in the ECM help construct a high interstitial pressure microenvironment in PDAC that is responsible for vascular resistance^76^ and can be used as a fuel source for cancer cells.^77^ Researchers have found that the accumulation of HA promotes macrophage infiltration and phenotypic differentiation to M2,^78^ and preclinical studies with hyaluronidase in combination with immunotherapy for PDAC have demonstrated efficacy in reducing the C-X-C chemokine receptor type 4 (CXCR4) immunosuppression signal axis expression in myeloid cells, decreasing CXCR4-expressing myeloid cells, and increasing T cell infiltration.^79^^,^^80^

#### Extracellular vesicles

PDAC cells secrete large amounts of circulating exosomes during carcinogenesis,^81^^,^^82^ and the effect of these exosomes, particularly encapsulated substances, on macrophages has become a popular research topic in recent years. PDAC cell-derived exosomes can transfer substances such as miR-155-5p^83^ and long non-coding RNA (lncRNA) FGD5-AS1^84^ to macrophages and polarize them to the M2-type. Annexin A1 (ANXA1) is externalized through exosomes, leading to the acquisition of a mesenchymal phenotype in both tumor and stromal cells; the complex ANXA1/EVs expedites macrophage recruitment and promotes M2 macrophage polarization.^85^ miR-155-5p transferred by the exosome attenuates AKT protein kinase signal transduction,^86^ and lncRNA FGD5-AS1 up-regulates and activates the STAT3/NF-κB signaling pathway in the macrophage, leading to M2 polarization. Interestingly, the production of PDAC-derived exosomes requires IL-6 induction, and the secretion of IL-6 by polarized macrophages promotes the expression of FGD5-AS1 in tumor cells, forming a positive feedback loop.^84^^,^^87^ Exosomes secreted by PDAC cells transfected with miR-155 and miR-125b reprogram M2-like macrophages to the M1 phenotype.^88^

In addition to the encapsulated substances, proteins on the membranes of extracellular vesicles also impact macrophage polarization. Exosomes secreted from the PDAC cell line AsPC-1 are rich in intercellular adhesion molecule-1 (ICAM-1), which increases M2-type markers through their interaction with exposed CD11c on macrophages.^89^ The arachidonic acid (AA) content in exosomes can also affect their fusion with macrophages, which has been shown in colorectal cancer.^90^ AsPC-1 cells contain high levels of AA. AsPC-1 exosomes fuse at a higher rate with macrophages than with exosomes from other PDAC cell lines, and macrophages treated with AsPC-1 exosomes show increased levels of M2-like surface markers that polarize to an immunosuppressive phenotype.^89^

#### Cancer-associated fibroblasts

As one of the most important stromal components in the TME, CAF-derived cytokines, chemokines, growth factors, and exosomes can induce immune evasion of cancer cells.^91^, ^92^, ^93^, ^94^ Pancreatic fibroblasts transduced by exogenous oncogenic KRAS activate inflammatory gene expression programs. Fibroblasts are the main source of cytokines that regulate macrophage polarization.^94^ CAF-derived CCL2 and hypoxia-inducible factor (HIF)-2 play a central role in macrophage recruitment.^95^^,^^96^ The loss of the tumor suppressor phosphatase and tensin homolog (PTEN) in CAFs results in increased phosphorylation of STAT3. Consequently, CAFs can increase CXC chemokine ligand 1 (CXCL1) and IL-6 secretion, promoting macrophage M2 polarization.^93^^,^^97^ On the other hand, IL-33, macrophage (M)-CSF, and miRNA-320a expressed mainly or exclusively by fibroblasts in a Kras^G12D^-dependent manner drive macrophage-mediated immune suppression.^92^^,^^94^^,^^98^ Meanwhile, MEKi^+^STAT3i-induced MSC-rich CAFs reprogramme M2 macrophages to restore the M1 phenotype and reshape the immune microenvironment.^99^

#### Hypoxia

PDAC is highly hypoxic,^100^ and the hypoxia-adaptive response mediated by HIFs renders it more aggressive and therapeutically resistant.^101^ A target of HIF-1, stromal cell-derived factor (SDF-1) produced by PDAC cells, recruits macrophages via the SDF-1/CXCR4 axis.^102^ HIF-1α can also promote tumor cells to secrete cytokines such as CCL2^103^ to recruit macrophages. HIF-2 is a protagonist that coordinates CAF-TAM crosstalk, which induces macrophage migration and M2 polarization in a paracrine fashion.^96^ Others like the hypoxia tumor-derived exosome miR-301A-3p is affected by *KRAS* to activate the PTEN/PI3Kγ signaling pathway in TAMs and induce M2 polarization of them.^104^

In a hypoxic environment, tumor cells, TAMs, and other cells undergo adaptive metabolic responses to meet the energy requirements for biosynthesis. There are metabolic differences between M1 and M2 macrophages, with the M1 type mainly dependent on glycolysis and the M2 type relying on the tricarboxylic acid cycle.^105^ In a hypoxic environment, lactic acid, a byproduct of glycolysis produced by tumor cells, induces the M2 polarization of TAMs. Notably, CCL18 secreted by M2-like macrophages can promote the paracrine induction of vascular cell adhesion molecule 1 (VCAM-1) in PDAC cells, whereas VCAM-1-induced lactate production from PDAC cells with enhanced aerobic glycolysis activates macrophages to an M2 phenotype, forming a positive feedback loop.^106^^,^^107^

## Tumor-associated macrophages in the tumor immune microenvironment

### Molecular characterization of immunosuppressive tumor-associated macrophages

**Figure 2:**
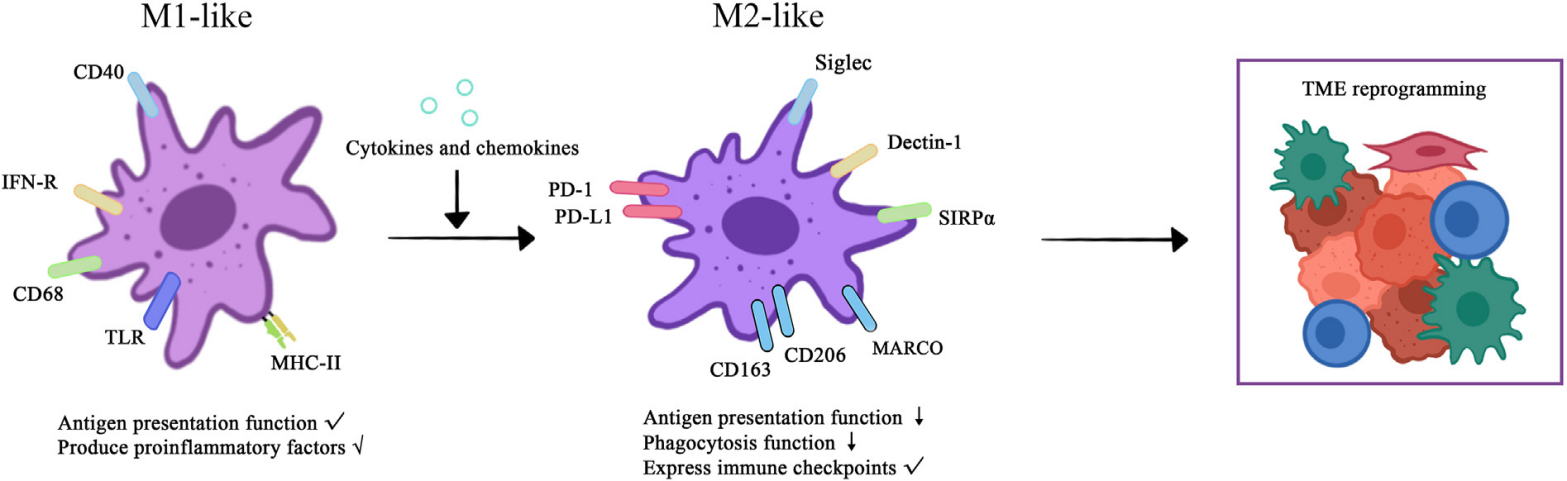
The different molecular characterization of M1 and M2 TAMs in PDAC. Different molecules serve both as markers for the two types of TAMs and shape functional differences between them. As the cancer progresses, changes in this molecular characterization are involved in shaping the immunosuppressive microenvironment. CD: Cluster of differentiation; IFN-R: Interferon receptor; M1: Classically activated macrophages; M2: Alternatively activated macrophages; MARCO: macrophage receptor with a collagenous structure; MHC-II: Major histocompatibility class II; PD-1: Programmed death 1; PDAC: Pancreatic ductal adenocarcinoma; PD-L1: Programmed death ligand 1; SIRPα: Signal-regulatory protein α; TAM: Tumor-associated macrophage; TLR: Toll-like receptor; TME: Tumor microenvironment.

During tumorigenesis and the development of PDAC, various surface molecules of TAMs adapt to the TME [Figure 2]. These molecules are hijacked by tumor cells, resulting in immunosuppression and the development of an immune escape system, which are major obstacles to immunotherapy.

#### Major histocompatibility class II

As an important antigen-presenting cell, MHC-II expression represents the antigen-presenting capacity of macrophages to trigger a CD4^+^ T cell response. During the transformation of normal pancreatic tissues into tumors, potential anti-tumor MHC-II^+^ macrophages decrease and even disappear, whereas tumor-promoting Arg-1^+^ macrophages are substantially increased.^12^ When the M2-like polarization of macrophages is inhibited, the expression of MHC-II on the surface of TAMs significantly increases.^108^ In addition, MHC-II expression levels are associated with the source. Embryonically derived TAMs express lower levels of MHC-II.^13^

#### Signal-regulatory protein α

SIRPα is an inhibitory receptor expressed on myeloid cells. SIRPα recognizes the ligand CD47, which is often overexpressed in cancer cells.^109^^,^^110^ Binding of CD47 to SIRPα suppresses the function of nonmuscle myosin IIA, which plays a pivotal role in phagocytosis.^111^ Many cancers evade attack by phagocytes through increasing CD47 expression, but it has been shown that the potent phagocytosis of low SIRPα-expressing macrophages when activated by pro-inflammatory factors is independent of CD47 expression.^111^ This suggests that blocking or depleting SIRPα on TAMs should be a key point for improving phagocytosis through the CD47-SIRPα axis.

#### Programmed cell death protein 1/programmed cell death ligand 1

PD-1 is often upregulated in activated T cells, leading to immune tolerance. Its ligand, PD-L1, is frequently overexpressed in tumor cells.^112^^,^^113^ PD-1 is also expressed on the surface of macrophages, which is related to phagocytic dysfunction, and PD-1^+^ TAMs express more of the M2-associated scavenger receptor CD206 and less MHC-II.^114^ The high expression of CXCR4 on TAMs is related to the upregulation of several immune checkpoints, including PD-1, PD-L1/programmed cell death ligand 2 (PD-L2), indoleamine 2,3-dioxygenase (IDO), T cell immunoglobulin, and the immunoreceptor tyrosine-based inhibitory motif (ITIM) domain (TIGHT), thus promoting immune evasion.^115^ PD-L2 is another receptor for PD-1 expressed on TAMs and is expressed at elevated levels in concert with IL-6 and IL-4.^116^

#### Dectin-1/galectin 9

Dectin-1 is highly expressed on the TAMs in PDAC and is capable of recognizing β-glucan polysaccharides from fungal cell walls^117^ and its ligand galectin 9 (gal-9), which is expressed in PDAC-infiltrating leukocytes and cancer cells.^118^ Dectin-1 deletion and blockade of gal-9 induce immunogenic reprogramming of TAMs toward M1-like cells, which elevates MHC-II and reduces the levels of CD206.^118^ High expression of Yes1-associated transcription regulator (YAP1) in PDAC tumor cells indicates poor prognosis, whereas ubiquitination and degradation of YAP1 reduce the expression of gal-9, inhibit M2 polarization of TAMs, and thus suppress immune escape.^119^

#### The Siglec family

Siglecs are immunoglobulin-like lectins that are mostly expressed by cells of the immune system and bind to sialic acid. ITIMs are the main components involved in the inhibition of immune receptors.^120^^,^^121^ Increased sialic acid in the TME of PDAC is recognized by Siglec-7 and Siglec-9 in myeloid cells and promotes the expression of PD-L1 and IL-10.^122^ Siglec-10 is also highly expressed on TAMs in PDAC and its interaction with CD24 in tumor cells promotes immune evasion by inhibiting phagocytosis.^110^^,^^123^ A recent study has revealed the presence of Siglec-15 in macrophages. Siglec-15 interacts with α-2,3 sialic acid expressed by PDAC cells, which stimulates spleen tyrosine kinase phosphorylation in TAMs, and upregulates *CCL2*, *CCL20*, *IL1β*, *CSF1*, *IL4I1*, *CXCL2*, *CD163*, and *CD206* expression. Moreover, Siglec-15^+^ TAMs tend to exhibit M2 phenotypes and are associated with poor prognosis in PDAC.^124^

#### Other members of the B7 family

The B7 family consists of cell-surface protein ligands that bind to lymphocyte receptors and positively or negatively regulate immune responses.^125^ B7-H3, B7-H4,^126^^,^^127^ and HHLA2 (B7-H7), as important inhibitory molecules, are all highly expressed in PDAC cells. In PDAC, B7-H3 positively correlates with B7-H4 or B7-H7 on TAMs, and a high expression of B7-H3 or HHLA2 in CD68^+^ TAMs indicates poor prognosis.^128^ B7-H5 is a molecule with limited expression in TAMs and an unidentified receptor^129^; however, high expression of B7-H5 induces a stronger immune response in PDAC.^130^

### Tumor-associated macrophages help shape the immune microenvironment of pancreatic ductal adenocarcinoma

**Figure 3:**
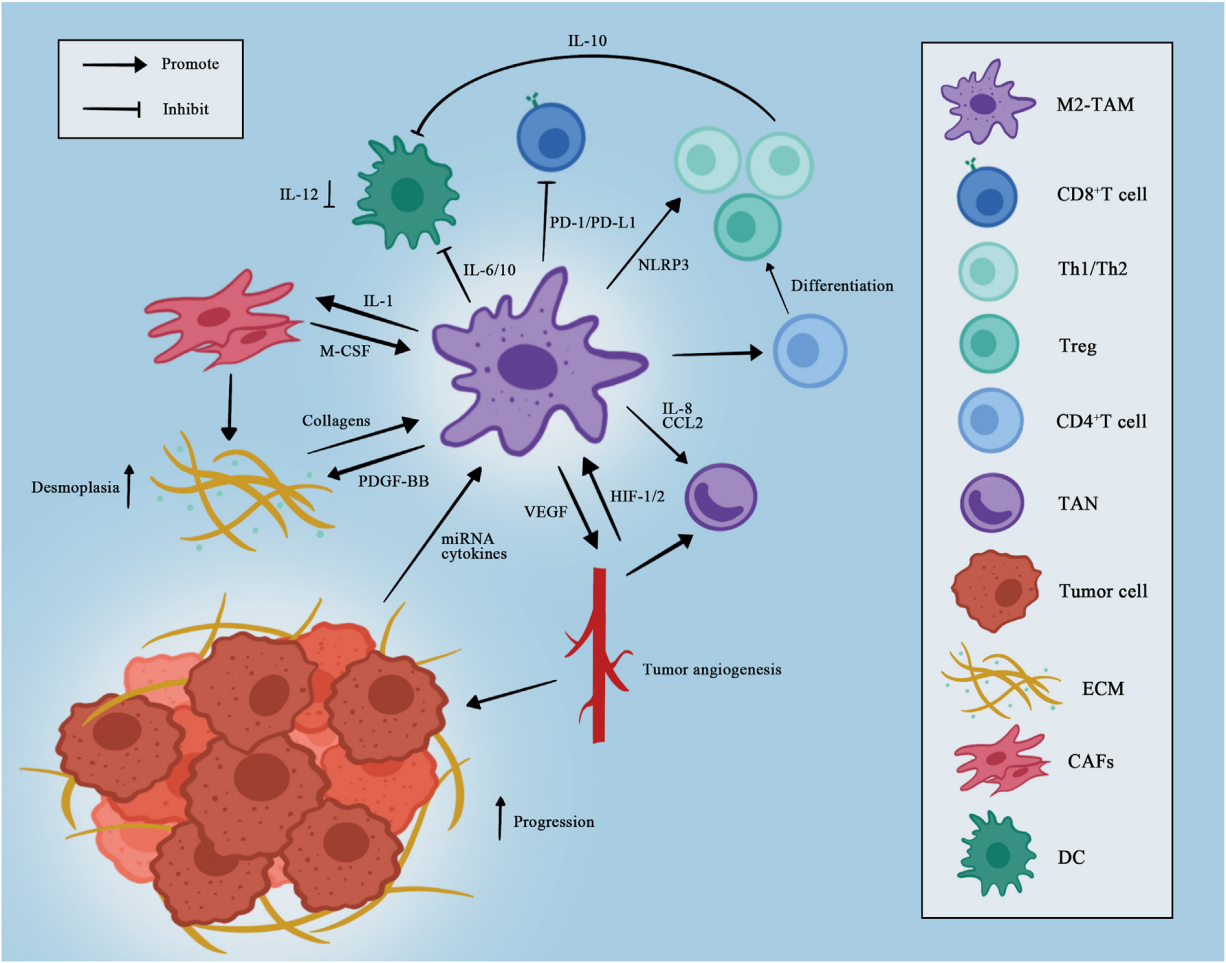
The immunosuppressive microenvironment with TAM as a center. TAM acts as a bridge to induce other cells to form an immunosuppressive phenotype. CAF; Cancer-associated fibroblast; CCL2: CC chemokine ligand 2; CD: Cluster of differentiation; DC: Dendritic cell; ECM: Extracellular matrix; HIF: Hypoxia-inducible factor; IL: Interleukin; M2: Alternatively activated macrophages; M-CSF: Macrophage colony-stimulating factor; miRNA: Microribonucleic acid; NLRP3: NOD-like receptor family pyrin domain containing 3; PD-1: Programmed cell death protein 1; PDGF-BB: Platelet-derived growth factor subunit B; PD-L1: Programmed cell death ligand 1; TAM: Tumor-associated macrophage; TAN: Tumor-associated neutrophil; Th: T helper; Treg: Regulatory T cell; VEGF: Vascular endothelial growth factor.

The immunosuppressive microenvironment of PDAC is a complex network composed of various immune cells, in which TAMs are the most abundant immune cells in the stroma,^12^ and their phenotypes and cytokine production play an indispensable role in shaping the immunosuppressive landscape [Figure 3].

#### Tumor-associated macrophages and cancer-associated fibroblasts

CAFs are widely accepted as one of the main regulators of macrophages. Macrophages also affect the functions of CAFs, and *vice versa*. As a cytokine reservoir, macrophages secrete IL-1α and IL-1β that influence the secretion of the thymic stromal lymphopoietin by CAFs.^131^ Under the stimulation of IL-33 secreted by iCAFs, TAMs highly express CXCL3, which induces the conversion of CAFs to myo-CAFs.^132^ Macrophage-secreted heterocellular oncostatin M (OSM) stimulates the expression of inflammatory mediators in CAFs, such as IL-6 family members, and CXC and CC-chemokines.^133^ A pan-cancer single-cell analysis showed that the prevalence of endothelial cell-mesenchymal transformation of CAFs may be related to the impact of proximal SPP1^+^ TAMs.^134^

#### Tumor-associated macrophages and T cells

Although infiltrating T cells constitute a small portion of the immune infiltration of PDAC, they are at the forefront of anti-tumor immunity, including CD8^+^ T cells, CD4^+^ T cells (T helper [Th] 1 and Th2 cells), Foxp3^+^ CD4^+^ T cells (Tregs), and Th17^+^.^135^ PD-1 and (CTLA4) on the surface of T cells are negative regulators of T cell immune function, and all their ligands can be found on TAMs. By reducing infiltrated TAMs in PDAC with clodronate liposomes, Yang et al^136^ found that CD8^+^ T cell infiltration increased, the number of CD4^+^ Foxp3^+^ Tregs decreased, and effector CD8^+^ T cells were spatially brought closer to tumor cells. TAMs can induce CD4^+^ T cells to differentiate into T cell subsets with tumor-promoting functions, such as Th2, Th17, and Treg populations, and in PDAC, this effect can be achieved through NOD-like receptor family pyrin domain containing 3 (NLRP3) signals in the TAMs via IL-1β.^137^ Moreover, tumor antigen presentation is required to induce monocyte differentiation into anti-tumor macrophages, promote Th1 cells, abrogate Treg cells, and mitigate CD8^+^ T cell exhaustion.^138^ Receptor-interacting serine/threonine-protein kinase 1 (RIP1) is upregulated in TAMs in PDAC. The inhibition of RIP1-expressing TAMs leads to an increase in cytotoxic T-cell activation and a pro-immune mixed phenotype of Th1/Th17.^139^

#### Tumor-associated macrophages and B cells

There was a large infiltration of B cells in the PDAC immunodynamic analysis.^12^ B cells can differentiate into plasma and memory B cells, which have either tumor-promoting or tumor-suppressing effects by producing antibodies and cytokines.^140^ In metastatic lesions of PDAC, macrophages culture with B cells which are recruited by tumor cells that secrete IL-18 and downregulate IFN-γ, MHC-II, and CD86.^141^ However, there is insufficient evidence to indicate whether TAMs affect B cell function. Crosstalk between TAMs and B cells in PDAC appears to exist; however, more evidence is required.

#### Tumor-associated macrophages and neutrophils

The proportion of neutrophils infiltrating PDAC is increased.^142^ Neutrophils are assumed to be both immunostimulatory (N1) and immunosuppressive (N2).^143^ Single-cell RNA sequencing (RNA-seq) analysis has shown that TAMs can attract neutrophils via the CCL13-CCR1, CCL3-CCR1, CCL3L3-CCR1, CXCL2-CXCR1, CXCL2-CXCR2, and CXCL8-CXCR2 axes,^144^ and the same applies for IL-8 and CCL2.^145^ IL-1β secreted by tumor cells promotes the infiltration of both M2-like TAMs and TANs into the PDAC immune microenvironment.^146^ Similar to macrophages, TANs can be induced to migrate by chemokines, such as GM-CSF, M-CSF, and CXCL1.^147^^,^^148^ N2-like neutrophils with immunosuppressive functions also express PD-L1 in PDAC.^149^ Hypoxia is a condition in which the interaction between TAMs and TANs is enhanced, as both can be recruited to the hypoxia site through HIF-1α.^103^ It can be speculated that the hypoxia in PDAC aggravates immunosuppression by inducting TANs and TAMs into immunosuppressive phenotypes and enhancing their mutual interactions.

#### Tumor-associated macrophages and dendritic cells

DCs are specialized antigen-presenting cells that capture antigens and present them as antigenic peptides to T cells of the immune system. During the development of PDAC, the expression levels of the maturation marker MHC-II and the co-stimulatory molecules CD40 and CD86 on tumor-associated DCs decrease, which can be attributed to an increase in IL-6,^150^ of which M2-like TAMs are an important source.^87^ IL-10 produced by TAMs and Treg inhibits the production of IL-12 by cDCs,^151^^,^^152^ which is a necessary co-stimulatory molecule to activate adaptive immune responses.

#### Tumor-associated macrophages and angiogenesis

TAM-induced angiogenesis is complex. TAMs often exhibit an M2-like phenotype and are key players in this process as they release vascular endothelial growth factor (VEGF), a potent stimulator of new blood vessel formation.^153^ TAMs also produce HIF-1α, a transcription factor of multiple angiogenic reaction genes such as IL-1β, IL-8, and MMPs.^154^ Moreover, TAMs interact with other cells in the TME, such as endothelial cells, to create a supportive niche for angiogenesis. TAMs secrete exosomes carrying miR-155-5p and miR-221-5p, which act on endothelial cells and promote angiogenesis in PDAC.^155^

#### Tumor-associated macrophages and desmoplasia

Desmoplasia is an important pathological feature that greatly influences tumorigenesis, metastasis, and drug resistance in PDAC.^156^ Platelet-derived growth factor-BB, IL-1, IL-6, and CCL2 chemokines secreted by TAMs help activate fibroblasts and promote desmoplasia.^157^^,^^158^ TAMs can also regulate ECM deposition by producing metalloproteinase 2 and MMPs, which play a significant role in the level of fibrosis in the TME.^157^

#### Tumor-associated macrophages and perineural invasion

Increased neural density is detected in PDAC.^159^ There is a trophic interaction between cancer cells and intratumoral nerves in the desmoplastic stroma.^160^ A recent study showed that sympathetic denervation by ablation increases the number of CD163^+^ macrophages in PDAC, increases the growth and spread of the tumor, and results in a worse prognosis.^161^ Another study showed that macrophage-related gene expression and F4/80-positive macrophages were increased in the left dorsal root ganglion of patients with neural invasion-induced allodynia in PDAC,^162^ suggesting that macrophages may be related to neural invasion in PDAC. However, no reports have indicated that the polarization of TAMs correlates with neural invasion.

## Targeting tumor-associated macrophages

In recent years, immunotherapy has mainly focused on immune checkpoint blockade (ICB), tumor vaccines, and cell therapy. However, none of these therapeutic approaches have demonstrated significant efficacy against PDAC. Because they play a core role in the immunosuppression of PDAC with diverse functions, TAMs seem to be ideal targets for immune microenvironment remodeling and have recently become a hot research topic [Table 1]. Broadly, there are three strategies for targeting TAMs, as explained in detail below.

**Table 1:** Ongoing clinical trials on macrophage-targets.

Compound	Clinical phase (status)	ClinicalTrial.gov ID	Combination drugs	Reference
**Chemokine inhibitors**				
BMS-813160 (CCR2/CCR5 antagonist)	Phase 1b/2 (completed)	NCT03184870	Nivolumab, nab-paclitaxel, gemcitabine, 5-FU, leucovorin, irinotecan	https://clinicaltrials.gov/study/NCT03184870?term=NCT03184870&rank=1
	Phase 1/2 (ongoing)	NCT03767582	Nivolumab, GVAX	https://clinicaltrials.gov/study/NCT03767582?term=NCT03767582&rank=1
TAK-500 (CCR2 and STING agonist)	Phase 1 (ongoing)	NCT05070247	Pembrolizumab	https://clinicaltrials.gov/study/NCT05070247?term=NCT05070247&rank=1
**Depletion agents**				
PLX3397 (CSF1R inhibitors)	Phase 1 (completed)	NCT02777710	Durvalumab	https://clinicaltrials.gov/study/NCT02777710?term=NCT02777710&rank=1
LY3022855 (anti-CSF1R antibodies)	Phase 1 (completed)	NCT03153410	Cyclophosphamide, GVAX, pembrolizumab	https://clinicaltrials.gov/study/NCT03153410?term=NCT03153410&rank=1
Cabiralizumab (anti-CSF1R antibodies)	Phase 1 (completed)	NCT02526017	Nivolumab	https://clinicaltrials.gov/study/NCT02526017?term=NCT02526017&rank=1
Lurbinectedin (DNA damage)	Phase 2 (ongoing)	NCT05229588	NA	https://clinicaltrials.gov/study/NCT05229588?term=NCT05229588&rank=1
**Reprogramming agents**				
BI 754091 (anti-CD47/SIRPα antibodies)	Phase 1 (ongoing)	NCT04752215	Ezabenlimab	https://clinicaltrials.gov/study/NCT04752215?term=NCT04752215&rank=1
CP-870, 893 (agonist anti-CD40 antibodies)	Phase 1 (completed)	NCT01456585	Gemcitabine	https://clinicaltrials.gov/study/NCT01456585?term=NCT01456585&rank=1
APX005M (agonist anti-CD40 antibodies)	Phase 1 (ongoing)	NCT02600949	Pembrolizumab, sotigalimab	https://clinicaltrials.gov/study/NCT02600949?term=NCT02600949&rank=1
	Phase 1b/2 (ongoing)	NCT05419479	Zimberelimab, domvanalimab	https://clinicaltrials.gov/study/NCT05419479?term=NCT05419479&limit=10&rank=1
RO7009789 (agonist anti-CD40 antibodies)	Phase 1 (completed)	NCT02588443	Nab-paclitaxel, gemcitabine	https://clinicaltrials.gov/study/NCT02588443?term=NCT02588443&limit=10&rank=1
Gedatolisib (PI3K/mTOR inhibitor)	Phase 1 (ongoing)	NCT03065062	Palbociclib	https://clinicaltrials.gov/study/NCT03065062?term=NCT03065062&limit=10&rank=1
Metformin	Phase 1 (ongoing)	NCT02336087	Gemcitabine, paclitaxel albumin-stabilized nanoparticle formulation	https://clinicaltrials.gov/study/NCT02336087?term=NCT02336087&limit=10&rank=1
	Phase 2 (completed)	NCT01666730	Oxaliplatin, leucovorin calcium, fluorouracil	https://clinicaltrials.gov/study/NCT01666730?term=NCT01666730&limit=10&rank=1
	Phase 2 (ongoing)	NCT04033107	Vitamin C	https://clinicaltrials.gov/study/NCT04033107?term=NCT04033107&limit=10&rank=1
**Macrophage cell therapy**				
CT-0508 (ACT-macrophages)	Phase 1 (ongoing)	NCT04660929	Pembrolizumab	https://clinicaltrials.gov/study/NCT04660929?term=NCT04660929&limit=10&rank=1

5-FU: 5-Fluorouracil; ACT: Adoptive Cell Transfer Therapy; CCR: Chemokine receptors; CD: Cluster of Differentiation; CSF1R: Colony-stimulating factor 1 receptor; mTOR: Mammalian target of rapamycin; PI3K: Phosphatidylinositol 3-kinase; SIRPα: Signal-regulatory protein α; STING: Stimulator of interferon genes.

### Blocking tumor-associated macrophages recruitment

CCL2/CCR2 signal transduction is the core axis promoting monocyte recruitment to the TME. CCR2 blockers can eliminate inflammatory monocytes and macrophages at the tumor site, enhance anti-tumor immunity, and reduce tumor growth.^61^^,^^163^ A clinical phase 1 trial targeting the CCL2/CCR2 signaling axis showed positive results, with an objective tumor response occurring in 49% and local tumor control in 97% of patients treated with the CCR2 antagonist PF-04136309 in combination with FOLFIRINOX chemotherapy.^164^ However, other reports suggest that the systemic release of IFN-γ and CCL2 by CD40 agonists synergistically alter the CCR2^+^ monocyte/macrophage infiltration of tumors, which may cause them to lack or lose the capacity to produce CCL2 and be unresponsive to anti-CD40 therapy,^165^ suggesting that blocking the CCL2/CCR2 axis to reduce TAMs recruitment may not be always positive. A CXCR1/2 inhibitor (ladarixin), which reduces the polarization and migration of M2-like macrophages, has been shown to enhance the anti-tumor effect of PD-1 inhibitors when combined^166^; however, no relevant clinical trials are underway.

### Tumor-associated macrophages depletion

Macrophage depletion therapy is still under investigation; however, Borgoni et al^167^ suggested that treatment with trabectedin, a cytotoxic drug that specifically targets TAMs, is an efficient strategy for obtaining an appropriate T cell anti-tumor immune response. Lurbinectedin (PM00183) is an anticancer drug that specifically eliminates TAMs in the TME by decoding the TNF-related apoptosis-inducing ligand (TRAIL) receptor^168^ and inducing apoptosis via the DNA damage response. The combined use of lurbinectedin and gemcitabine results in a complete tumor response by inducing an increase in gemcitabine-mediated DNA damage. Moreover, lurbinectedin induces TAM depletion, leading to cytidine deaminase downregulation in PDAC tumors, which in turn induces an increase in gemcitabine-mediated DNA damage.^169^ Targeting the CSF1-CSF1 receptor (CSF1R) axis has a comprehensive effect on the tumor immune microenvironment. SOM230, a long-acting cyclohexapeptide somatostatin analog, has been approved for the treatment of neuroendocrine tumors. When administered to PDAC, it reduces the production of CSF-1 by CAFs and decreases the activities of CAFs and TAMs in the stroma, thereby alleviating chemotherapy-induced (gemcitabine) immunosuppressive stroma reshaping.^170^ Not only does it deplete TAMs, but it also reprograms the remaining TAMs into the M1 type, enhances their antigen-presenting function, and reinforces the efficacy of T cells, among other things.^108^ In animal experiments, the CSF1R selective inhibitor AZD7507 enhanced T cell immune response and reduced the proliferation of malignant cells by targeting macrophages in PDAC.^171^ In a combination therapy based on the PDAC vaccine (GVAX), anti-PD-1, and anti-CSF1R antibodies, PD-1^+^ T cells can be converted into CD137^+^-activated effector T cells^172^ after the addition of anti-CSF1R antibodies, thereby increasing the hypersensitivity responses to autologous tumor cells of PDAC patients.^173^^,^^174^

### Tumor-associated macrophages reprogramming

TAMs can be reeducated to reverse their immunosuppressive M2 phenotype to a tumor-killing M1 phenotype. TAM re-education can be achieved through sphingomyelin synthase 2 inhibitor, which down-regulates the expression of IL-4Rα and CSF1R to reduce M2 polarization.^175^ The immunomodulator pomalidomide induces NF-κB activation, which subsequently inhibits IFN regulatory factor 4, a transcription factor that promotes M2 polarization, thus transforming TAMs into pro-inflammatory populations.^176^^,^^177^

The CD47/SIRPα axis is an important regulatory signal for inhibiting the innate immune function of macrophages. Preclinical studies have shown that the inhibition of CD47 on the surface of PDAC cells can effectively regulate the phagocytosis of macrophages,^178^^,^^179^ thereby indirectly completing in-service education for macrophages. Anti-CD47 antibody treatment seems to be effective because the survival time can be significantly improved in combination with other immunotherapies (such as HAC, a PD-L1 blocker).^180^ However, it increases the risk of side effects owing to the widespread expression of CD47.^178^^,^^181^ A phase 1 clinical trial of a novel bispecific antibody, PT886, that targets claudin 18.2 and CD47 is ongoing (NCT05482893). Upon radiotherapy (RT)-mediated activation, TAMs acquire pro-inflammatory features and enhance the tumoricidal function of the microenvironment. Depletion of SIRPα on TAMs enhances the efficacy of RT, with RT-mediated activation followed by potent pro-inflammatory features and immunogenic antigen presentation by SIRPα-deficient macrophages within tumors that confer a tumoricidal microenvironment highly infiltrated by tumor-specific cytotoxic T cells, natural killer (NK) cells, and inflammatory neutrophils.^182^ This suggests that a combination with RT and SIRPα antibody has a certain application prospect. The scavenger receptors CD206 and CD163 are highly expressed in PDAC TAMs.^183^ The synthetic peptide RP-182 can induce a change in the conformation of CD206 and reprogram M2-like TAMs into M1-like TAMs, which effectively inhibits the growth of solid tumors.^184^ The Siglecs family has also been described previously. But at present, in PDAC, besides CD47/SIRPα with the ongoing clinical trials, clinical studies of the above two kinds of myeloid checkpoints are still lacking.

Monoclonal CD40 agonist antibodies (e.g., CP-870, 893, APX005M, RO7009789, and SEA-CD40) have been evaluated in clinical trials.^185^ Several agonistic CD40 monoclonal antibodies (mAbs) are currently being used in combination with immunotherapy or chemotherapy. STING agonists (like cobimetinib and dimethylxanthenone acetic acid [DMXAA]) can increase the expression of co-stimulatory molecules in DCs, reprogram TAMs, and enhance anti-tumor immunity. In preclinical trials, single or combined treatment with autophagy (mefloquine) and agonistic CD40 mAbs could both effectively change the tumor structure.^186^^,^^187^ TLR agonists can also potently activate the immune response of macrophages.^120^ The combination of the TLR9 agonist IMO-2125 with anti-PD-1 therapy improves the anti-tumor activity of the PDAC immune microenvironment.^188^ The application of the TLR3 agonist CMP-001 and TLR9 agonist SD-101 in PDAC has entered the clinical trial stage.

Owing to their unique metabolic pattern, TAMs can be reprogrammed by blocking certain metabolic pathways.^120^ For example, the glycolysis inhibitor, 2-deoxy-d-glucose (2-DG) significantly changes PDAC immunosuppression.^189^ A phase 1 dose-escalation trial of 2-DG combined with chemotherapy for advanced solid tumors provided the recommended dose of 2-DG^190^; however, there have been no clinical studies on 2-DG in PDAC. Metformin is a glucose metabolism intervention agent used for the treatment of diabetes, and its use significantly improves the clinical outcome and survival rate of patients with Smad4-deficient PDAC.^191^ Metformin can reprogram TAMs into an anti-tumor phenotype.^192^ However, in a double-blind, randomized, placebo-controlled phase 2 trial, metformin did not improve the outcomes in patients with advanced pancreatic cancer treated with gemcitabine and erlotinib.^193^ Thus, the application of biguanides to PDAC requires further investigation. Sirolimus, a mammalian target of rapamycin (mTOR) inhibitor, can change the immune microenvironment through metabolic reprogramming to enhance the glycolysis of M2 macrophages, thereby improving the blocking effect of PD-L1.^194^

TiE2 is a receptor tyrosine kinase, and patients with PDAC with higher TiE2^+^ TAM frequencies have shown a greater risk of tumor development as a metastatic disease,^195^^,^^196^ whereas the TiE2 inhibitor rebastinib has been shown to be effective in reducing the growth and metastasis of pancreatic neuroendocrine tumors in mice.^195^ Tumor-targeting EnGeneIC Dream Vector nanocells, a potent cytoimmuno therapeutic, showed anti-tumor function by polarizing M1 TAMs and activating NK cells, concurrently producing a Th1 cytokine response.^197^ The positive effects of TAM reprogramming in preclinical trials suggest that regulation of TAMs can ameliorate the immunosuppressive microenvironment of PDAC. However, the functional diversity of TAMs and the complex crosstalk of the immune microenvironment of PDAC determine the difficulty of a single immune-targeted therapy, and previous clinical drug studies have shown that a single drug does not meet clinical needs well. In the future, multi-target combined immunotherapy will continue to be the development trend for the adjuvant therapy of PDAC. However, there is a long way to go in its clinical applications.

Macrophages play a dual role in shaping the immune microenvironment of PDAC cells. They can act as torchbearers, promoting tumor immunity, or as accomplices undermining anti-tumor immunity. Macrophages are integral to the construction of the complex immune microenvironment network of PDAC and are influenced by various components within this microenvironment. Many of these regulatory mechanisms remain unexplored and the insights provided in this study represent only the tip of the iceberg of this intricate immune network. Therefore, future research should adopt a broader perspective and fully consider the intricate interconnections within the TME to overcome the challenges of pancreatic cancer immunotherapy.

## Concluding remarks and perspective

The immunosuppressive microenvironment of PDAC is an important intrinsic factor in its refractory nature to immunotherapy. Together with tumor and immune cells, TAMs participate in various processes that shape the immunosuppressive microenvironment, making them popular therapeutic targets for PDAC. Clinically, PDACs present with various biological behaviors, including conventional chemoresistance, chemosensitivity, local invasiveness, and small primary lesions with a heavy metastatic burden. However, the role of TAMs in the development of these clinical phenotypes has not been determined. In preclinical studies, targeting TAMs by reducing recruitment or controlling the polarization direction has achieved promising results. However, these results are often unexpected in clinical trials. This may be due to differences in human and mouse macrophages, dissimilarity in macrophage-induced polarization *in vivo* and *in vitro*, and the heterogeneity of human PDAC tumors. Therefore, the origin, differentiation, and functional heterogeneity of TAMs in PDAC require further investigation. TAMs interact with other microenvironmental cells to form a large immune network and research on this topic is still evolving. Elucidating these relationships and underlying mechanisms is crucial for the development of TAM-targeting therapy.

## Authors contribution

Runjie Liu: conceptualization, writing – original draft; Jianang Li: writing – original draft, writing – review & editing; Liang Liu: conceptualization, supervision, writing – review & editing; Wenquan Wang: conceptualization, supervision, writing – review & editing; Jinbin Jia: conceptualization, supervision – review & editing. All the authors critically revised and approved the final version of the manuscript.

## Ethics statement

None.

## Declaration of generative AI and AI-assisted technologies in the writing process

The authors declare that generative artificial intelligence (AI) and AI assisted technologies were not used in the writing process or any other process during the preparation of this manuscript.

## Funding

This work was supported by grants from the Shanghai Municipal Health Commission Health Industry Clinical Research Project (No. 201940019), 10.13039/501100001809National Natural Science Foundation of China (Nos. 82273382, 82272929, 82103409, 81972218, 81972257, and 81827807), Shanghai ShenKang Hospital Development Centre Project (No. SHDC2020CR2017B), Program of Shanghai Academic/Technology Research Leader (No. 23XD1400600), 10.13039/501100002858China Postdoctoral Science Foundation (No. 2021M690037), Shanghai Sailing Program (No. 21YF1407100), Beijing Xisike Clinical Oncology Research Foundation (Nos. Y-2022METAZQN-0003, Y-HR2022MS-0251, and Y-HR2022QN-0085), and Science and Technology Planning Project of Yunnan Province (No. 202305AF150148).

## Data availability statement

The datasets used in the current study are available from the corresponding author on reasonable request.

## Conflict of interest

The authors declare that they have no known competing financial interests or personal relationships that could have appeared to influence the work reported in this paper.
